# Rescued Chondrogenesis of Mesenchymal Stem Cells under Interleukin 1 Challenge by Foamyviral Interleukin 1 Receptor Antagonist Gene Transfer

**DOI:** 10.3389/fphar.2017.00255

**Published:** 2017-05-09

**Authors:** Nicole Armbruster, Jennifer Krieg, Manuel Weißenberger, Carsten Scheller, Andre F. Steinert

**Affiliations:** ^1^Institute for Virology and Immunobiology, University of WuerzburgWuerzburg, Germany; ^2^Department of Orthopaedic Surgery, Klinik König-Ludwig-Haus Würzburg – Center for Musculoskeletal Research, University of WuerzburgWuerzburg, Germany

**Keywords:** mesenchymal stem cell, chondrogenesis, pellet culture, foamy virus, virus vectors, IL1RA, interleukin 1 receptor antagonist, arthritis

## Abstract

**Background:** Mesenchymal stem cells (MSCs) and their chondrogenic differentiation have been extensively investigated *in vitro* as MSCs provide an attractive source besides chondrocytes for cartilage repair therapies. Here we established prototype foamyviral vectors (FVV) that are derived from apathogenic parent viruses and are characterized by a broad host range and a favorable integration pattern into the cellular genome. As the inflammatory cytokine interleukin 1 beta (IL1β) is frequently present in diseased joints, the protective effects of FVV expressing the human interleukin 1 receptor antagonist protein (IL1RA) were studied in an established *in vitro* model (aggregate culture system) of chondrogenesis in the presence of IL1β.

**Materials and Methods:** We generated different recombinant FVVs encoding enhanced green fluorescent protein (EGFP) or IL1RA and examined their transduction efficiencies and transgene expression profiles using different cell lines and human primary MSCs derived from bone marrow-aspirates. Transgene expression was evaluated by fluorescence microscopy (EGFP), flow cytometry (EGFP), and ELISA (IL1RA). For evaluation of the functionality of the IL1RA transgene to block the inhibitory effects of IL1β on chondrogenesis of primary MSCs and an immortalized MSC cell line (TERT4 cells), the cells were maintained following transduction as aggregate cultures in standard chondrogenic media in the presence or absence of IL1β. After 3 weeks of culture, pellets were harvested and analyzed by histology and immunohistochemistry for chondrogenic phenotypes.

**Results:** The different FVV efficiently transduced cell lines as well as primary MSCs, thereby reaching high transgene expression levels in 6-well plates with levels of around 100 ng/ml IL1RA. MSC aggregate cultures which were maintained in chondrogenic media without IL1β supplementation revealed a chondrogenic phenotype by means of strong positive staining for collagen type II and matrix proteoglycan (Alcian blue). Addition of IL1β was inhibitory to chondrogenesis in untreated control pellets. In contrast, foamyviral mediated IL1RA expression rescued the chondrogenesis in pellets cultured in the presence of IL1β. Transduced MSC pellets reached thereby very high IL1RA transgene expression levels with a peak of 1087 ng/ml after day 7, followed by a decrease to 194 ng/ml after day 21, while IL1RA concentrations of controls were permanently below 200 pg/ml.

**Conclusion:** Our results indicate that FVV are capable of efficient gene transfer to MSCs, while reaching IL1RA transgene expression levels, that were able to efficiently block the impacts of IL1β *in vitro*. FVV merit further investigation as a means to study the potential as a gene transfer tool for MSC based therapies for cartilage repair.

## Introduction

The repair capacity of articular cartilage is very limited, among others due to the lack of vascularization that could provide progenitor cells to the injured tissue (Caplan et al., 1997; Patra and Sandell, 2012; Orth et al., 2014). Therapies so far are based on the implantation of autologous chondrocytes at the site of the lesion, or marrow-stimulating approaches for the recruitment of bone-marrow derived mesenchymal stem cells (MSCs) (Brittberg et al., 1994; Steinert et al., 2007). MSCs can be isolated readily from several sources, like for instance bone marrow, blood, and mesenchymal tissues (Nöth et al., 2010). Moreover, they are able to self-renew and differentiate into multiple tissues, which makes *ex vivo* expanded MSCs an attractive alternative cell source to chondrocytes (Pittenger et al., 1999). MSCs are already intensively investigated and applied in clinical trials for regenerative therapies in the musculoskeletal system (Steinert et al., 2012). However, such demands failed so far and did not result in the desired sustained regeneration of hyaline cartilage *in vivo*, as the newly formed tissue resulted widely in fibrocartilage like structures (Steinert et al., 2007; Orth et al., 2014). A major problem seems thereby the insufficient delivery of soluble factors for driving the chondrogenic differentiation of the transplanted cells *in vivo* (Steinert et al., 2007; Madry et al., 2011). To overcome this problem, gene transfer technologies have been intensely used to study diverse candidate genes such as bone morphogenic proteins, Indian hedgehog (Ihh) and the SOX (SRY [sexdetermining region Y]-related HMG [high-mobility group] box) family of transcription factors for modulation of the chondrogenic differentiation *in vitro* (Ikeda et al., 2004; Steinert et al., 2009; Haddad-Weber et al., 2010). Among the currently used viral vector systems, are human immunodeficiency virus (HIV)-based orthoretroviral-, Moloney leukaemia virus (MLV)-, adenoviral-, and recombinant adenoassociated virus (rAAV) vectors (Gouze et al., 2002; Palmer et al., 2003; Pagnotto et al., 2007; Steinert et al., 2008; Frisch et al., 2015). Single stranded and self-complementary rAAV vectors are among the most promising vectors for gene therapy so far (Kay et al., 2009; Watson et al., 2013; Cucchiarini and Madry, 2014; Rey-Rico et al., 2015).

Here we studied the use of foamyviral vectors (FVV) for gene delivery to human MSCs. FVV are derived from apathogenic parental viruses and might be a safe and efficient alternative for stable gene transfer (Rethwilm, 2007; Armbruster et al., 2014). They are naturally self-inactivating and have a big packaging capacity due to their large (∼13 kb) proviral genome (Lindemann and Rethwilm, 2011). As therapeutic target in this setup we choose delivery of the interleukin 1 receptor antagonist protein (IL1RA), as the inflammatory cytokin interleukin 1β (IL1β) is highly expressed in diseased and injured joints and a known mediator of cartilage breakdown, synovial inflammation, as well as a known inhibitor of chondrogenesis (Wehling et al., 2009; Kraus et al., 2012; Martínez de Albornoz Torrente and Forriol, 2012; Schett et al., 2016).

## Materials and Methods

### Recombinant DNA

**FIGURE 1:**
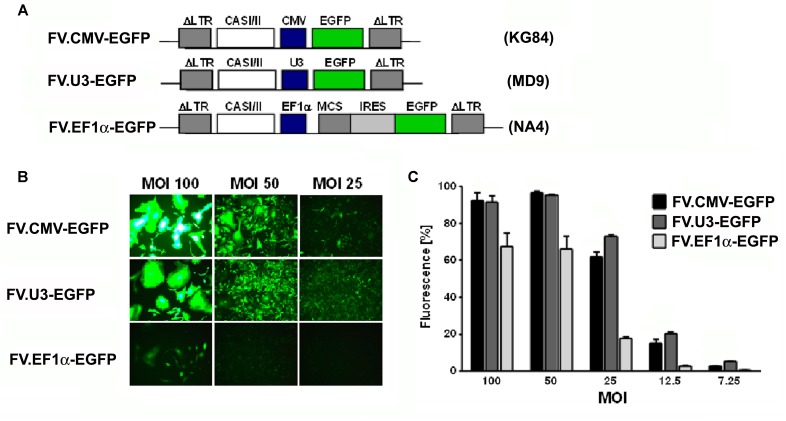
**Vector optimization.** Part 1: Promoter study. **(A)** Schematic illustration and designation of foamyviral vectors (FVV) expressing enhanced green fluorescent protein (EGFP) with different promoters. The expression of the EGFP genes were under the control of the constitutively active cytomegalovirus immediate early promoter, the SFFV U3 promoter, or the human elongation factor (EF) 1α promoter, and the corresponding foamyvirus vectors (FVV) (and vector plasmids) were disignated FV.CMV-EGFP (KG84), FV.U3-EGFP (MD09), and FV.EF1α-EGFP (NA4). IRES (internal ribosomal entry site), CASI/II (cis-acting sequences; required for vector transfer), MCS (multiple cloning site), LTR (long terminal repeat). **(B)** Fluorescence microscopy of HT1080 transduced fibroblasts 3 days post-transduction with the different FVV at different multiplicities of infection (MOI). 100× magnification. **(C)** Quantification of transduction rates analyzed by EGFP expression in FACS analyses 3 days post-transduction at different vector dilutions (MOI). MOCK controls revealed no EGFP positive cells (data not shown). Data are shown as mean + SD with *n* = 2 experiments and three replicates per condition.

**FIGURE 2:**
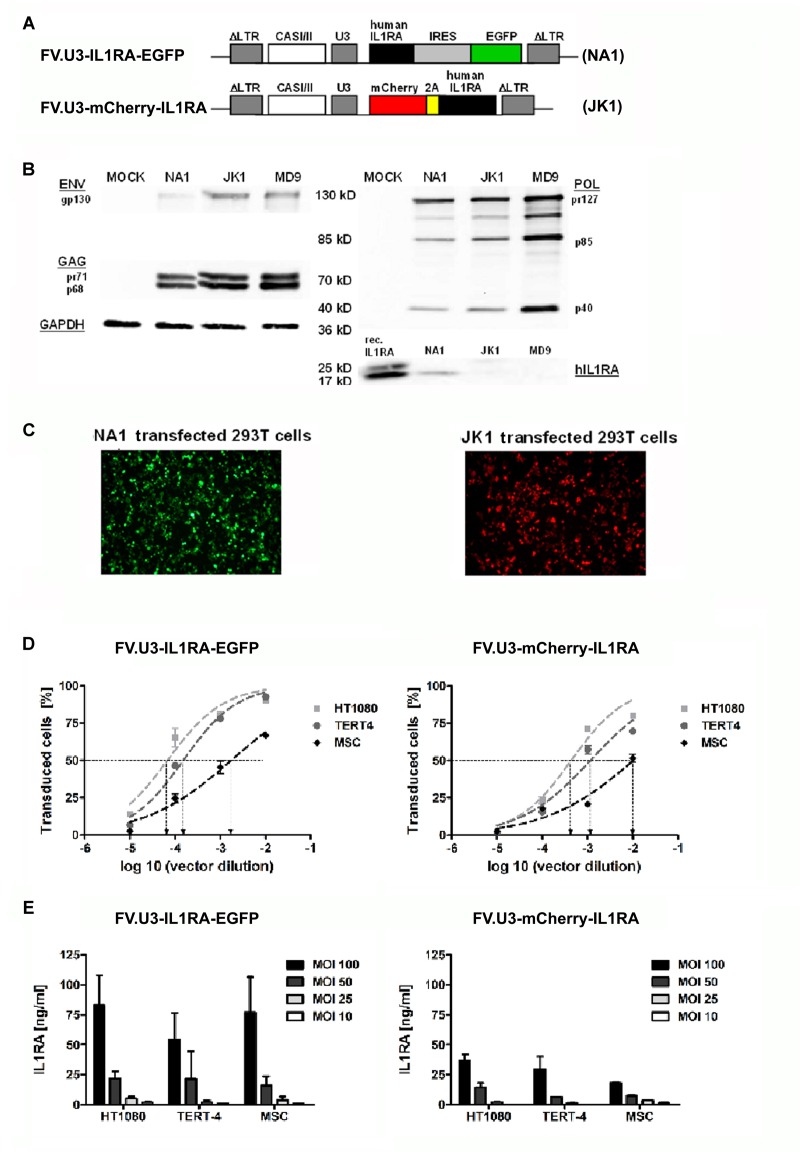
**Vector optimization.** Part 2: Vector verification study. **(A)** Schematic illustration of the prototype foamy virus vectors under control of the SFFV U3 (SFFV-U3) promoter expressing the therapeutic gene interleukin 1 receptor antagonist (IL1RA) along with a marker gene via two different modes. The FVV were designated (with corresponding name of the vector plasmid) FV.U3-IL1RA-EGFP (NA1) and FV.U3-mCherry-IL1RA (JK1). In the bicistrionic FV.U3-IL1RA-EGFP vector, the internal ribosomal entry site (IRES) of the encephalomyocarditis virus allows the co-expression of IL1RA and EGFP from a single promoter and two open reading frames (ORF). In the FV.U3-mCherry-IL1RA vector, the linking of mCherry and IL1RA with the self-cleaving small foot-and-mouth disease virus (FMDV) 2A peptide results in expression of the two proteins derived from a single ORF and also a single promoter. **(B)** Exemplary western blot analysis. Detection of prototype foamy virus (PFV) GAG, POL, and ENV proteins as well as IL1RA in cells cotransfected with the respective FVV plasmid and GAG-, POL-, and ENV-encoding constructs. Glyceraldehyde-3-dehydrogenase (GAPDH) served as loading control. Recombinant human hIL1RA was used as positive control for the IL1RA blot. **(C)** Fluorescence microscopy of 293T cells 24 h after transfection with the respective vector plasmids NA1 (left; green fluorescence) and JK1 (right; red fluorescence). 100× magnification. **(D)** Transduction rates following infection of different mesenchymal cell types with FV.U3-IL1RA-EGFP (left) and FV.U3-mCherry-IL1RA (right). HT1080 fibroblast cell line, TERT4 mesenchymal stem cell (MSC) line, and primary MSCs were incubated with dilution series of vector preparations for dosing and calibration of vector solutions. EGFP+ cells were determined after 48 h by flow cytometry. Using a sigmoidal curve function, CCID50 (cell culture infectious dose 50 – the vector dilution at which the half-maximal transduction rate was visible) concentration values were determined by nonlinear regression. **(E)** Levels of secreted human IL1RA measured by a specific ELISA in cell culture supernatants from transduced cells with different doses of FV.U3-IL1RA-EGFP (left) and FV.U3-mCherry-IL1RA (right). MOCK controls (data not shown) were below the detection limit. Data are shown as means + or ± SD with *n* = 3 experiments and triplicate measurements per condition. MOI = multiplicity of infection.

All used foamy vector (FV) plasmids were derived from the plasmid MD9 (Heinkelein et al., 2002; Peters et al., 2005; Wiktorowicz et al., 2009) that expresses the enhanced green fluorescent protein (EGFP) markergene driven by the spleen focus forming virus (SFFV)-U3 promoter (**Figure 1A**). The FV plasmids NA1 and KG84 (**Figures 1A**, **2A**) were already described earlier (Gartner et al., 2009; Armbruster et al., 2014). For cloning of the FV vector plasmid construct NA4 that expresses EGFP via an internal ribosomal entry site (IRES) of the encephalomyocarditis virus driven by the human elongation factor 1α (EF1α) promoter (Uetsuki et al., 1989), EF1α was PCR amplified with specific primers using the pEF-GW-51-lacZ plasmid as PCR template (Gateway Vektor System, Invitrogen) and inserted into the pBF014 plasmid via the AfeI and AscI restriction site (Armbruster et al., 2014) (**Figure 1A**). The plasmid JK1 was derived from a pTW01 based FV plasmid, namely pTW22, coding for mCherry with a fused 2A cleavage site and EGFP (Ryan et al., 1991; Wiktorowicz et al., 2009). The EGFP was replaced with the PCR amplified human IL1RA cDNA, which was inserted via RsrII and SalI (**Figure 2A**). The structure of each plasmid was verified by restriction mapping and sequencing prior to use. FV vectors were abbreviated with the respective promoter and insert (followed by the name of the corresponding vector plasmid), namely FV.CMV-EGFP (KG48), FV.U3-EGFP (MD9), FV.EF1α-EGFP (NA4), FV.U3-IL1RA-EGFP (NA1), FV.U3-mcherry-IL1RA (JK1) (illustrated in **Figures 1A**, **2A**).

### Foamyviral Vector Production

Viral vector stocks were generated by transient transfection of HEK 293T cells as descriebd earlier (Stange et al., 2005; Wiktorowicz et al., 2009; Armbruster et al., 2014). Briefly, 8 × 10^6^ cells were seeded on 10 cm-dishes and were transfected the next day using 20 μg of total plasmid DNA with the following ratio 10:5:1:1 of vector plasmid and packaging plasmids (pCZIgag2, pCZIpol, and pCZ-HFVenvEM002) using polyethylenimine (Polysciences) (Stange et al., 2005). MOCK controls were also performed, using a transfection mix lacking the env plasmid and empty pcDNA to adjust the DNA amount. One day after transfection cellular transcription was induced by addition of 10 mM Na-butyrate for 8 h. After 2 days the supernatants were harvested, passed through a 0.45-μm filter (Millipore) and stored in aliquots at –80°C. Vectors were optionally concentrated to higher titers by centrifugation (polyallomer tubes, 12000 g, 4°C, 2 h). The infectious titer of the FVV preparations were determined using vector dilutions and an immunofluorescence assay as previously described (Peters et al., 2008). Vector preparations were adapted to 3.5 × 10^7^ infectious particles/ml and stored at –80°C. (Peters et al., 2008). FVV and transduced cells were handled in the laboratories of the Department of Virology, University of Wuerzburg under appropriate biosafety level 2 conditions according to German law (Gentechniksicherheitsverordnung-GenTSV).

### Cell Culture, Vector Transfer, Transgene Expression

The vector-containing supernatant was assayed functionally by transfer to 1 × 10^4^ cells by using different doses of infectious virus, as given per multiplicities of infection (MOI) in the respective experiments. For characterization studies the expression of EGFP was monitored by fluorescence-activated cell sorting (FACS) using a FACSCalibur flow cytometer (Beckton Dickinson) if not otherwise mentioned 72 h after transduction. Levels of cell culture infectious dose 50 (CCID_50_) were determined using the GraphPad prism 4.0 software (GraphPad Software). The vector transfer assays were done at least three times with different plasmid preparations. As recipient cells the human fibroblastic cell line HT1080, the human TERT4 MSC line and primary human MSCs were used (Abdallah et al., 2005). Primary MSCs were obtained from bone marrow of several human donors undergoing total hip replacement surgery after informed consent and as approved by the by institutional review board of Wuerzburg University. MSCs were isolated by adherence of cells harvested from the patient’s spongiosa to plastic and maintained as described previously (Nöth et al., 2002).

For evaluation of IL1RA transgene expressions conditioned media from cell cultures were collected over a 24-h period and stored at –20°C. The human IL1RA concentrations were measured by ELISA (DuoSet Elisa Development Quantikine kit, R&D Systems). The minimum detectable dose of the human IL1RA ELISA is 6.26 pg/ml according to the manufacturer. All measurements were performed in triplicates.

### Aggregate Culture

Primary MSCs and the mesenchymal TERT4 cells were transduced with FVVs at 1000 MOI in T-125 flasks at ∼40% confluency to obtain transduction efficiencies of around 50%, which were confirmed by detection of green fluorescence. Three days after transduction EGFP+ cells were sorted using a FACSDiVa (Beckton Dickinson). Selection efficiency was determined to be approximately 99 %. After one week, expansion sorted primary MSCs and mesenchymal TERT4 cells were trypsinized and placed in aggregate cultures as described earlier (Johnstone et al., 1998; Penick et al., 2005). Briefly, cells were distributed in 15 ml polypropylene tubes (Falcon) at a concentration of 3 × 10^5^ cells/pellet in 500 μl medium [serum-free DMEM, 37.5 mg/ml ascorbate, 1 mM pyruvate, 10^-7^ M dexamethasone, 1% ITS (insulin, transferrin, and selenous acid containing culture supplement), all Sigma] to promote aggregate formation. To induce chondrogenesis 10 ng/ml recombinant TGFβ1 protein (R&D Systems) was added. Further, aggregates were additionally supplemented with 5 ng/ml IL1β (R&D Sytems) to inhibit chondrogenesis. The aggregates were cultured at 37°C, 5% CO2 and formed spherical pellets within 24 h. Changes of media were performed every 2 to 3 days. The aggregates were harvested after 21 days for further analysis. At least three different aggregates per group and bone marrow preparations from three different preparations were analyzed if not otherwise mentioned. MSC passages for aggregate cultures ranged from passage 4-6.

### Cell Proliferation and Glycosaminoglycan Assay

Cell proliferation in aggregates was assessed by quantitative detection of adenosine 5′-triphosphate (ATP), which correlates with the number of viable cells present, using the CellTiter-Glo^®^ Luminescent Cell Viability Assay (Promega) according to the manufacturer’s instructions. Briefly, pellets were homogenized mechanically using a pellet pestle and mixed with 100 μl of CellTiter-Glo^®^ reagent (CellTiter-Glo^®^ substrate + CellTiter-Glo^®^ buffer). After incubation for 10 minutes at room temperature luminescence was measured using a plate-reading luminometer.

For analysis of glycosaminoglycan (GAG) content, aggregates were washed with phosphate buffered saline (PBS), digested with 200 μl of papain digest solution (1 μg/ml, Sigma), and incubated for 16 hours at 65°C. Samples were stored at –20°C. Total GAG content was measured by reaction with 1,9-dimethylmethylene blue using the Blyscan^TM^ Sulfated Glycosaminoglycan Assay (Biocolor Ltd) as directed by the supplier. For normalization, DNA content of aggregates was also determined fluorometrically using the Quant-iT^TM^ PicoGreen^®^ kit as directed by the supplier (Invitrogen).

### Histological and Immunohistochemical Analyses

For histological analyses, aggregates were fixed in 4% paraformaldehyde for one hour afterward dehydrated in graded alcohols, embedded in paraffin and sectioned to 5 μm. Representative sections were stained using haematoxylin and eosin (H&E) for evaluation of cellularity and alcian blue (Sigma) for the detection of matrix proteoglycan.

For immunohistochemical analyses sections were washed for 20 min in Tris-buffered saline (TBS) and incubated in 5% bovine serum albumin (BSA) (Sigma). Following washing in TBS, sections were pre-digested with pepsin at 1 mg/ml in Tris–HCl (pH 2.0) for 15 min at room temperature. Sections were then incubated overnight at 4°C with a monoclonal anti-COL II primary antibody (diluted in 0.5% BSA, Acris Antibodies GmbH) for detection of collagen type II (Collagen II). Immunostaining was visualized by treatment with peroxidase-conjugated antibodies (Dako) followed by diaminobenzidine staining (DAB kit; Sigma). The slides were finally counterstained with hemalaun (Merck).

### Immunoblotting

Analysis of viral protein expression was done essentially as described previously (Peters et al., 2005). In brief, cell lysates were prepared using lysis buffer (RIPA plus protease inhibitors). Viral proteins were probed with anti-Gag (Heinkelein et al., 2002), anti-Pol and anti-Env (Imrich et al., 2000) mouse monoclonal antibodies after separation in sodium dodecyl sulfate-containing 8% polycrylamide gels and semi-dry blotting onto Hybond ECL membranes (Pharmacia). Protein bands were detected using horseradish-coupled secondary antibodies (Dako) and employing the ECL detection system (Pharmacia). For detection of human ILRA protein, the antibody SC-25444 from Santa Cruz was used (1/200 dilution). As loading control glyceraldehyde-3-phosphate dehydrogenase (GAPDH) was used (0.5 μg/ml, IMG-5143A, Imgenex).

### Total RNA Extraction, Semi-quantitative, and Real-Time RT-PCR

Total RNA from cultured cells was isolated using Trizol (Invitrogen) and reverse transcribed using the iScript^TM^ cDNA Synthesis Kit (BioRad). For quatitative polymerase chain reaction (qPCR) analyses, the iCycler iQ system (BioRad) with QuantiFast SYBR Green PCR Master Mix was used. PCR was conducted in tripicates for each sample. Isoform-specific primers were used for the expression of human IL1RA (Sigma–Aldrich) and βActin (QuantiTect Primer Assay, Qiagen) was used for normalization. The primer sequences were as follows: human IL1RA forward 5′-ggcctccgcagtcacctaatcactct-3′, reverse 5′-ttgacacaggacaggcacat-3′. The amplified transcripts were quantified using the comparative ΔΔCT method (Pfaffl; et al., 2002).

### Statistical Analyses

The numerical data were expressed as mean values plus standard deviation (SD). All experiments were performed in triplicates on *n* = 2–8 different samples as indicated in the respective experiments. Where indicated, numerical data were subjected to variance analysis (one or two factor ANOVA) and statistical significance was determined by student’s *t*-test with *p* < 0.05 considered statistically significant, *p* < 0.01 considered very significant and *p* < 0.001 considered extremely significant. All measurements were done with a minimum of three technical replicates if not otherwise mentioned.

## Results

### Foamyviral Vector Optimization

We first aimed to compare three different promoters in their ability to drive the EGFP expression in FVV constructs by transducing the human fibrosarcoma cell line HT1080 (**Figure 1**). For this, the EGFP transgene in the FVV was under the control of either the constitutively active SFFV-U3 promoter, the cytomegalovirus immediate-early promoter (CMV) or the human EF1α promoter via an IRES site (**Figure 1A**). Accordingly, the cells were cultured and transduced with different vector doses. Three days thereafter fluorescence microscopy and FACS analysis revealed a dose-dependent effect of EGFP expression by all FVV types at different dilutions (**Figure 1C**). The two viral promoters were thereby similarly strong, whereas the EF1α promoter/IRES construct showed less EGFP+ cells, and fluorescence also appeared weaker under the fluorescence microscope (**Figures 1B,C**). To obtain a more detailed and representative comparison, the majority of the transduced cells should contain at best one viral integration per cell. At the low FV concentrations used, namely MOI 7.25, 12.5, and 25 in our experiments, FV fabricated from the SFFV-U3 construct were stronger than the CMV and EF1α promoter construct vectors (**Figure 1C**). We further verified a centrifugation protocol which we used for the concentration of our vector preparations. Fluorescence microscopy and CCID50 determination of the standard control vector 3 days after the respective transduction, revealed a 26-fold concentration factor compared to the uncentrifuged FVV preparation (data not shown).

As recently published, we exploited already FVV carrying the SFFV-U3 promoter for driving the transgene expression in rat knee joints (Armbruster et al., 2014). We therefore were interested in comparing our FV.U3-IL1RA-EGFP (NA1) in which an IRES site allows the co-expression of IL1RA and EGFP from the SFFV-U3 promoter, with a new FVV called FV.U3-mCherry-IL1RA (JK1) (**Figure 2A**). The FV.U3-mCherry-IL1RA vector is based on a FVV with a 850 bp shorter *cis*-acting sequence (CAS) that was previously characterized and is sought to improve the safety and packaging capacitiy of available FVV (Wiktorowicz et al., 2009). Further the linkage of mCherry and IL1RA with the self-cleaving small foot-and-mouth disease virus (FMDV) 2A peptide in the FV.U3-mCherry-IL1RA construct results in expression of the two proteins derived from a single open reading frame (ORF) (**Figure 2A**). Western blot analysis of 293T whole cell lysates transfected with the respective FVV plasmid and gag-, pol-, and env-encoding constructs showed the presence of the FV precursor and processed proteins (**Figure 2B**). The IL1RA levels from the FVV (NA1 and JK1) transfected cells were unexpectedly low compared to the loaded recombinant hIL1RA control. Two faint bands, corresponding to a 25 kD glycosylated and a 17 kD unglycosylated IL1RA form were visible within the JK1 transfected cells, whereas one 17 kD IL1RA band was detectable with the NA1 transfected cell lysate. EGFP and mCherry expression of the two constructs were further verified by fluorescence microscopy 24 h after transfection (**Figure 2C**).

We next determined CCID50 values (the vector dilution at which the half-maximal transduction rate was visible) of the FV.U3-IL1RA-EGFP (NA1) and FV.U3-mCherry-IL1RA (JK1) vector (**Figure 2D**). In order to compare the two different vectors, HT1080 fibroblasts, mesenchymal TERT4 cells and primary MSCs were incubated with a dilution series of the same vector preparation. As depicted in **Figure 2D**, primary MSCs were less susceptible (CCID50: JK1 = 0.01; NA1 = 0.003) to FVV transductions than TERT4 (CCID50: JK1 = 0.001; NA1 = 0.0002) and HT1080 cells, that were more susceptible (CCID50: JK1 = 0.0004; NA1 = 0.00007). This corresponded for instance to a 24- and 38-fold-lower susceptibility for primary MSCs compared to HT1080 cells for the FV.U3-IL1RA-EGFP and FV.U3-mCherry-IL1RA vector, respectively. Overall the the FV.U3-IL1RA-EGFP CCID50 values were about one log ratio smaller than the FV.U3-mCherry-IL1RA values, implying a better transduction efficiency of the FV.U3-IL1RA-EGFP vector. In parallel we analyzed the secreted IL1RA levels in the cell culture supernatants from the transduced cells with a specific ELISA (**Figure 2E**). Here we obtained corresponding results to the FACS analysis with a higher IL1RA secretion from the FV.U3-IL1RA-EGFP than from the FV.U3-mCherry-IL1RA transduced cells. Interestingly we also found that although we obtained lower transduction rates with MSCs compared to HT1080 and TERT4 cells, the secreted IL1RA levels from MSCs were comparable to the two cell lines for both FVV vectors. For instance the FV.U3-IL1RA-EGFP transductions with an MOI of 100 resulted in mean IL1RA amounts of 83, 54, and 77 ng/ml for HT1080, TERT4 and MSCs, respectively (**Figure 2E**).

### FV.U3-IL1RA-EGFP Vector Verification

**FIGURE 3:**
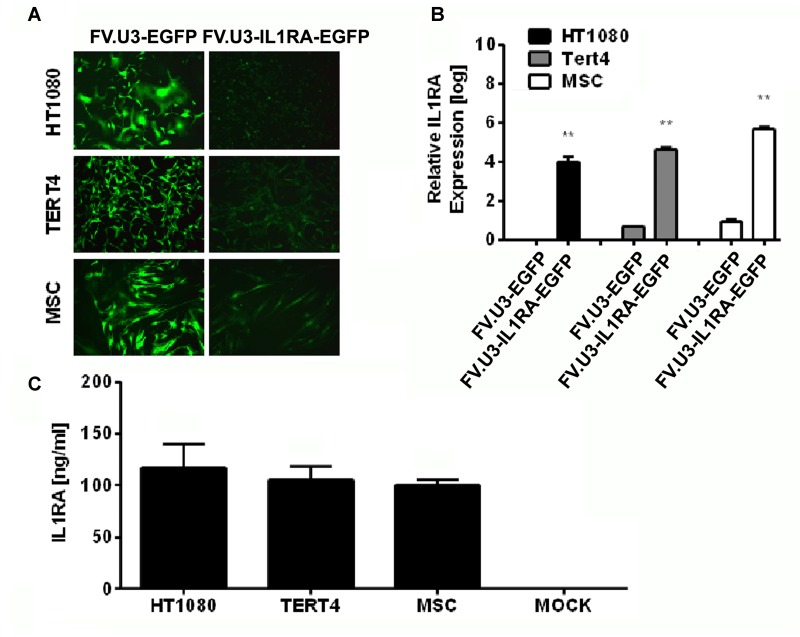
**Foamy vector (FV)-mediated gene delivery to human mesenchymal cell lines and primary MSCs.** **(A)** Fluorescence microscopy of fibroblastic HT1080 cell line, MSC cell line TERT4 and primary MSCs (MSC) transduced with a high dose of 1000 MOI of FV.U3-EGFP (left) or FV.U3-IL1RA-EGFP (right) vectors at 3 days post-transduction. 100× magnification. **(B)** Real-time PCR analysis of human IL1RA mRNA expression after transduction with FV.U3-EGFP and FV.U3-IL1RA-EGFP vectors. The values were calculated using the ΔΔCT method as described earlier, with FV.U3-EGFP or FV.U3-IL1RA being the treatment groups, MOCK the control group, IL1RA the target gene and β-Actin the reference gene. Values represent means + SD of three independent experiments (*n* = 3) per group at day 3 three of culture. Levels of significance compared to the controls (Student’s *t*-test) are indicated by asterisks (^∗^*p* < 0.05, ^∗∗^*p* < 0.01, ^∗∗∗^*p* < 001). **(C)** Levels of secreted human IL1RA in cell culture supernatants were detected by a specific ELISA (MOI 1000). Data are shown as mean + SD with *n* = 2 experiments and three technical replicates per condition.

**FIGURE 4:**
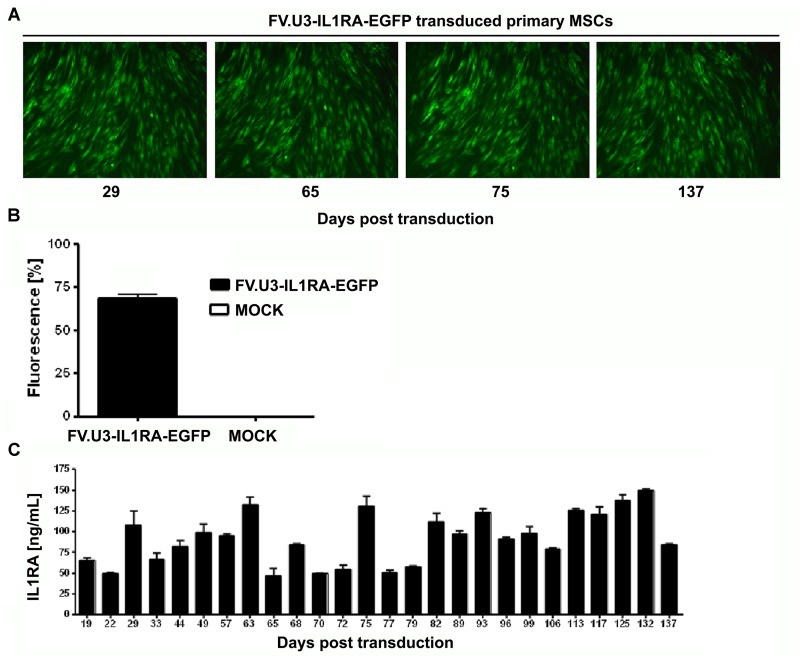
**Long-term FV-mediated transgene expression in primary MSCs.** **(A)** Fluorescence microscopy of human marrow-derived primary MSCs transduced with FV.U3-IL1RA-EGFP at 1000 MOI. Images taken over time are shown at 100× magnification. **(B)** Final FACS analysis of green fluorescence at 137 days post-transdcution of the FV.U3.IL1RA-EGFP cultures compared to MOCK controls. Values are given as mean + SD of *n* = 3 measurements. **(C)** IL1RA levels in the supernatant were measured by ELISA over time, and 24-hour-accumulations were analyzed. The experiment shown was done with MSCs from one donor and values represent means + SD from triplicate measurements per timepoint.

We further verified the FV.U3-IL1RA-EGFP (NA1) vector in more detail using different cell lines and primary cells (**Figures 3**, **4**). Fluorescence microscopy 3 days after the respective transductions displayed as already shown, that HT1080, TERT4, and MSCs are highly susceptive to gene delivery with FV.U3-IL1RA-EGFP (NA1) and FV.U3-EGFP (MD9) vectors (**Figure 3A**) at 1000 MOI. To avoid possible variability of expression due to the choice of promoter at low FVV doses, a maximum dose of 1000 MOI was chosen for this experiment. Due to the IRES a weaker EGFP expression from the FV.U3-IL1RA-EGFP vector compared to the FV.U3-EGFP construct was observed. This was not surprising as differences in expression levels due to the use of an IRES from the up- or downstream gene were already reported (Houdebine and Attal, 1999; Mizuguchi et al., 2000).

The numbers of EGFP+ cells in the respective cultures transduced with different dilutions of the FV.U3-IL1RA-EGFP vector were quantified using FACS analyses 3 days after vector exposure, and revealed a dose-dependent effect of EGFP expression at different dilutions (not shown). FVV-mediated expressions of the human IL1RA transgene in the different cells were analyzed by real-time RT-PCR on RNA (**Figure 3B**), and by ELISA at the protein level (**Figure 3C**). Production of IL1RA mRNA in the respective cultures 3 days after transduction with FV.U3-IL1RA-EGFP or FV.U3-EGFP vectors relative to MOCK controls revealed that only the FV.U3-IL1RA-EGFP cultures expressed IL1RA mRNAs at high levels (**Figure 3B**). Levels of secreted IL1RA protein in cell culture supernatants conditioned by the different cell types transduced with the same MOI (1000) of FV.U3-IL1RA-EGFP were measured by ELISA and displayed significantly elevated levels of IL1RA expression, with mean values of 105, 117, and 100 ng/ml, respectively (HT1080, TERT4, and MSCs) (**Figure 3C**).

Mesenchymal progenitor cells present an attractive source as an alternative to chondrocytes in cell-based approaches for cartilage repair. After verifying the ability of the FV.U3-IL1RA-EGFP to transduce primary MSCs we analyzed the long-term mediated transgene expression of this vector in monolayer cultures. MSCs were transduced in monolayer, FACS sorted for EGFP+ cells and placed in 6-well plates with medium exchange every 3–4 days. Fluorescence microscopy at several time points over 137 days in culture exposed a sustained EGFP expression over time (**Figure 4A**). Also, a final FACS analysis at day 137 showed that 89% of cells were viable and 68 % of the living cells were EGFP+ (**Figure 4B**). Most strikingly an ELISA analysis over time confirmed IL1RA mean protein levels between 46 and 149 ng/ml over the 137 days in culture, which represents accumulated IL1RA protein concentration in the supernatant over a 24 h period at the respective timepoints. The sustained FVV mediated transgene expression of an anti-inflammatory transgene seems interesting, as MSC *in vitro* cultures are known to adopt a state of permanent cell-cycle arrest and are thought to undergo cellular senescence almost from the moment of *in vitro* culturing (Bonab et al., 2006; Steinert et al., 2007, 2012).

### Aggregate Cultures of FVV Transduced Mesenchymal TERT4 Cells and Primary MSCs

**FIGURE 5:**
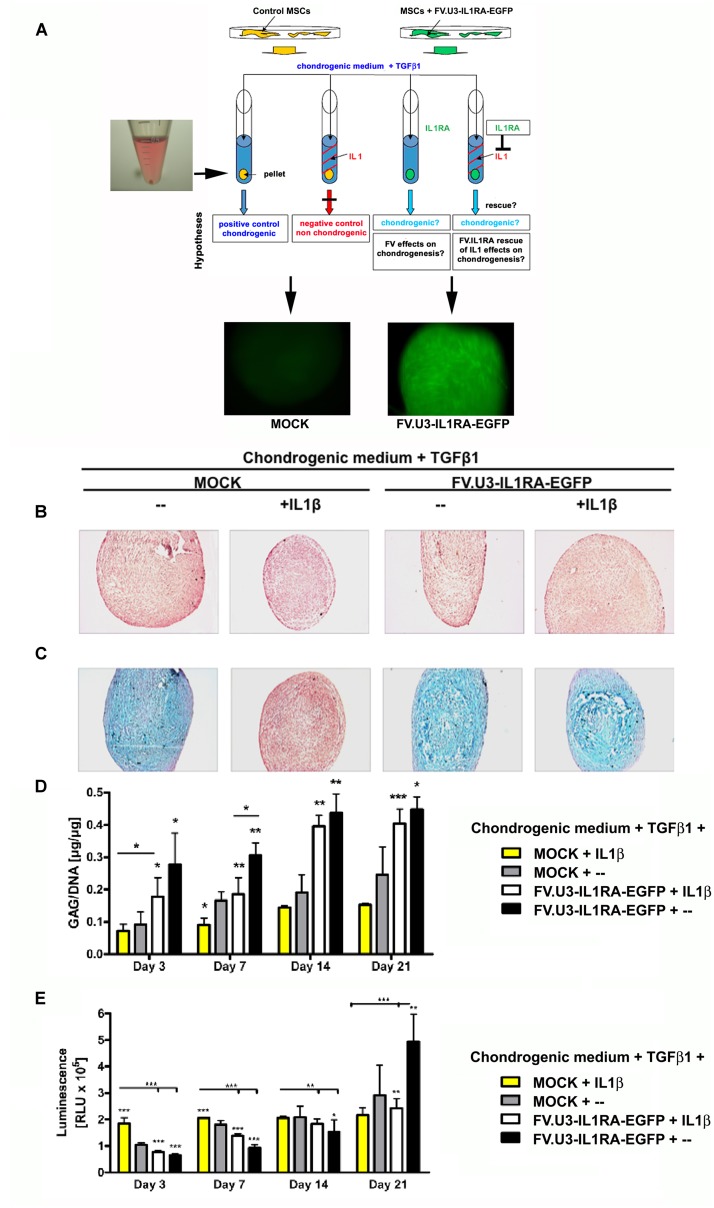
**Chondrogenesis assay using the MSC cell line TERT4.** **(A)** Schematic overview of the different treatment groups. Monolayer cultures were MOCK treated or FV.U3-IL1RA-EGFP transduced at 1000 MOI, FACS sorted for EGFP+ cells, and placed into aggreagte cultures with 3 × 10^5^ EGFP+ cells/pellet. Aggregates were treated with TGF1β (10 ng/ml) for chondrogenic induction. For chondrogenesis inhibition media were supplemented with recombinant human IL1β (5 ng/ml). **(B)** Representative paraffin sections with H&E staining for evaluation of cellularity and cell morphology after 21 days of pellet culture. **(C)** Alcian blue staining staining for detection of matrix proteoglycan after 21 days of pellet culture. **(D)** Time-course analysis of glycosaminoglycan (GAG) content normalized to DNA. **(E)** At distinct time points cell proliferation was quantified using the ATP cell proliferation assay. ATP = adenosine 5 triphosphate. The data represent mean values + SD from two experiments and three aggregates per condition and time point. Statistical significance was given by asterisks relative to MOCK controls without IL1β supplementation (MOCK + –) and by bars and asterisks upon multiple comparisons between groups (^∗^*p* < 0.05, ^∗∗^*p* < 0.01, ^∗∗∗^*p* < 001).

We next aimed to evaluate the functionality of the IL1RA transgene with studying its ability to block the inhibitory effects of IL1β on chondrogenesis of primary MSCs and the TERT4 MSC line in 3D pellet cultures. Therefore, primary MSCs and the TERT4 MSC cell line were stimulated along the chondrogenic pathway with a standard dose of 10 ng/ml TGFβ1 (Johnstone et al., 1998; Steinert et al., 2009). The cells were maintained following their transduction with FV.U3-IL1RA-EGFP at 1000 MOI in monolayer as pellet cultures in standard chondrogenic media in the presence or absence of 5 ng/ml IL1β (**Figure 5A**). The schematic of the experimental set-up using pellet cultures is depicted in **Figure 5A**, and a macroscopic image of a TERT4 control pellet in a 15-ml-conical-tube after three days is shown on the left, and representative images upon fluorescence microscopy at 100× of MOCK control and FV.U3-IL1RA-EGFP aggregates are shown at the bottom (**Figure 5A**).

After three weeks of TERT4-aggregate culture, the pellets were harvested and analyzed for chondrogenic phenotypes (**Figures 5B,C**). MOCK control TERT4 aggregates supplemented with TGFβ1 showed a significant chondrogenic response shown by a strong metachromic staining for matrix proteoglycans with alcian blue (**Figure 5C**). On the other hand, aggregates treated with IL1β, showed an inhibited chondrogenesis with negative alcian blue staining and smaller appearing pellets (**Figures 5A,B**; left images). Moreover, the transduction with the FV.U3-IL1RA-EGFP and respective IL1RA expression was able to rescue the inhibiting effect of IL1β on the chondrogenic differentiation, shown by restored positive alcian blue stainings (**Figures 5A,B**; right images).

In addition, for a quantitative comparison of the extracellular matrix synthesis between the different treatment groups over time, GAG levels in the TERT4 pellets were determined (**Figure 5D**). Pellets treated with IL1β showed significantly decreased GAG contents compared to the other treatment groups. Besides, already at day 3 significantly elevated GAG synthesis levels in the FV.U3-IL1RA-EGFP and FV.U3-IL1RA-EGFP + IL1β treated pellet groups became apparent and lasted over the 3 weeks of culture (**Figure 5D**). At distinct time points we also quantified the cell proliferation using an ATP cell proliferation assay. The IL1β treated pellets showed the highest proliferation levels among the treatment groups at day 3, but then stayed approximately equal over time. Within all the other treatment groups the cell proliferation levels increased gradually over time (**Figure 5E**).

**FIGURE 6:**
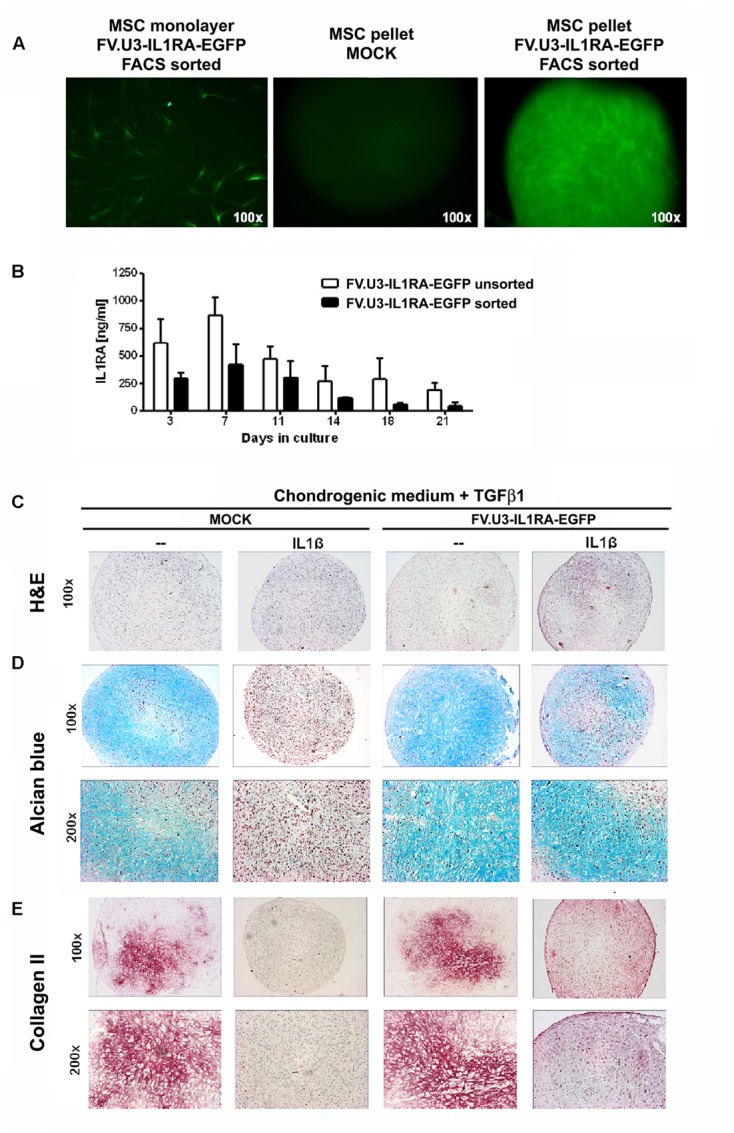
**Chondrogenesis assay using primary MSCs.** **(A)** Fluorescence microscopy of FV.U3-IL1RA-EGFP transduced monolayer MSCs 3 days post-transduction and aggregates of untreated control (MOCK) and FV.U3-IL1RA-EGFP transduced aggregates. **(B)** Effect of cell sorting on the transgene expression. MSCs were transduced in monolayer with FV.U3-IL1RA-EGFP and half of the cells were FACS sorted (EGFP) 2 days later. After 1 week expansion, both experimental groups were placed in aggregate cultures (3 × 10^5^ cells/pellet) and supernatants were harvested at distinct time points. Levels of secreted human IL1RA were measured by a specific ELISA. Data are represented as mean + SD. MSCs from two donors with three aggregates per experimental group and time point were analyzed. C-E) MOCK controls and FV.U3-IL1RA-EGFP transduced EGFP+ FACS sorted MSCs were placed in aggregate cultures and treated with TGFβ1 (10 ng/ml) for chondrogenic induction. For chondrogenesis inhibition media were supplemented with recombinant IL1β (5 ng/ml). **(C)** Representative paraffin sections with H&E staining for evaluation of cellularity and cell morphology after 21 days of pellet culture. **(D)** Alcian blue staining for detection of matrix proteoglycan. **(E)** Immunohistochemical analyses for detection of the cartilage matrix protein collagen type II (Collagen II) after 3 weeks. Collagen II staining with positive staining regions that appear red. 100× and 200× magnifications were used.

In another experiment a similar experimental set-up (see **Figure 5A**) has been applied to aggregate cultures of primary MSCs that were genetically modified with FV.U3-IL1RA-EGFP (NA1) at 100 MOI or not (MOCK controls) (**Figure 6**). First, we analyzed the EGFP expression levels of primary MSCs after transduction with FV.U3-IL1RA-EGFP and EGFP-FACS-sorting in monolayer and aggregate culture compared to MOCK controls and representative images upon fluorescence microscopy are presented in **Figure 6A**.

Then we compared the IL1RA expression levels of primary MSC aggregates that were transduced with FV.U3-IL1RA-EGFP in monolayer and either FACS sorted or not (**Figure 6B**). After 1 week expansion both experimental groups were placed in aggregate cultures and supernatants were harvested at distinct time points. Within both groups, the transgene expression levels peaked at day 7 and decreased thereafter over time (**Figure 6B**). Possibly due the FACS sorting procedure, the sorted pellets showed thereby at each time point approximately markedly lower IL1RA amounts compared to the levels of the unsorted pellets. Transduced unsorted MSC pellets reached very high IL1RA levels with a peak of 1087 ng/ml after day 7, followed by a decrease to 194 ng/ml after day 21 (**Figure 6B**), while IL1RA concentrations of controls were permanently below 200 pg/ml (not shown).

After 21 days of primary MSC-aggregate culture, the pellets were harvested and analyzed for chondrogenic phenotypes (**Figures 6C–E**). MOCK control pellets supplemented with TGFβ1 showed a significant chondrogenic response shown by increased aggregate size and a strong positive proteoglycan staining with alcian blue, while aggregates treated with IL1β, negative alcian blue staining and smaller appearing pellets (**Figures 6C,D**; left panels). Notably, the transduction with FV.U3-IL1RA-EGFP was able to rescue the inhibiting effect of IL1β on the chondrogenic differentiation, revealed by restored positive alcian blue stainings (**Figures 6C,D**; right panels).

We further conducted the immunohistochemical analysis of collagen type II (Collagen II) in primary MSC aggregates which is the predominant collagen type in hyaline cartilage (**Figure 6E**). MSC aggregates that were stimulated along the chondrogenic pathway with TGF-β1 revealed a strong Collagen II staining that was abrogated with IL1β supplementation (**Figure 6E**; left images). On the other hand, transduction with the FV.U3-IL1RA-EGFP was able to rescue the inhibiting effect of IL1β on the chondrogenic differentiation, shown by a restored Collagen II staining (**Figure 6E**; right images).

## Discussion

Foamyvirus vectors are derived from foamy viruses, also known as spumaretroviruses, that constitute a subfamily of retroviruses (Rethwilm, 2007). In our recent work, we evaluated prototype FVV for an *ex vivo* gene delivery approach in rat knee joints (Armbruster et al., 2014). Here we explored their patterns of transgene expression in different mesenchymal cell lines, especially we were interested in studying their ability to also transduce primary human MSCs. We assessed and optimized FVV transduction efficiencies using different vector modifications and examined the functionality of the expressed IL1RA transgene in an *in vitro* model of chondrogenesis.

In a first attempt we compared three FVV constructs with different ubiquitous promoters. We found that the two viral promoters, CMV and SFFV-U3, were significantly stronger in HT1080 cells compared to the cellular EF1α promoter (**Figure 1**). We acknowledge that this was not a fair comparison, as the construct containing the EF1α promoter also had an IRES site as opposed to the other vector constructs included in this study, which is often responsible for attenuated expression of genes downstream of the IRES. As it is known that promoters can vary considerably in their strength between different cell types, and as the CMV promoter was reported recently as very weak in rat MSCs (Qin et al., 2010; McGinley et al., 2011), we further focused on the already *in vivo* verified SFFV-U3 promoter constructs. Interestingly the EF1α promoter has been reported to be superior compared to the other promoters tested (CMV and PGK (phosphoglycerate kinase)) within lentiviral constructs in experiments using rat MSCs (Xia et al., 2007; McGinley et al., 2011). For clinical applications, the use of endogenous promoters seems favorable as viral promoters are often susceptible to transcriptional silencing in particular cell types (Bestor, 2000). The FVV constructs using the human EF1α promoter could therefore be interesting for future studies on human primary MSCs. Furthermore, FVV could be effectively concentrated around 25-fold by ultracentrifugation in polyallomer tubes (data not shown) confirming utility of this vector system similar to previous results (Vassilopoulos et al., 2001; Vassilopoulos and Rethwilm, 2008; Leurs et al., 2003).

Further, we compared for the co-expression of the IL1RA and EGFP or mCherry transgene, by using an IRES or a self-cleaving 2A peptide FVV construct for dual transgene expression (Donnelly et al., 2001; Kim et al., 2011). The comparison between the two FVVs revealed higher IL1RA expression levels and transduction efficiencies with the IRES FVV construct FV.U3-IL1RA-EGFP (NA1) compared to the 2A peptide FVV construct FV.U3-mCherry-IL1RA (JK1) (**Figure 2**). Although the IRES construct seemed superior, the vector with the shorter CAS possesses a higher safety profile and might be more suitable for future clinical applications (Wiktorowicz et al., 2009). Despite the advantage of a shorter viral sequence in the FV.U3-mCherry-IL1RA (JK1) vector, also the replacement of the IRES with the self-cleaving 2A peptide might provide further benefits, hence the IRES can be unreliable and doesn’t provide equal levels of expression of the separated genes, with the downstream sequence being usually translated at much lower levels than the upstream sequence (Trichas et al., 2008). Our western blot analysis of lysates from 293T cells transfected with FVV plasmids showed overall unexpectedly faint IL1RA signals, that unfortunately did not allow us to study uncleaved bands of IL1RA-mCherry fused protein amounts (**Figure 2B**). Here, cell lysates and supernatants from FV-transduced cells could provide a more detailed western blot pattern in the future. However, despite the interesting technical specifics detailed above in co-expressing two transgenes, it remains to be seen whether such dual transgene expression approaches via FVV will be of any clinical relevance at all in the future.

Although FVV cannot be pseudotyped (Wu and Mergia, 1999), a very broad tropism makes them a promising tool for gene delivery to various cells (Plochmann et al., 2012). As already mentioned, because of the capability of MSCs to differentiate into several lineages, including chondrocytes, adipocytes and osteoblasts, as well as their role in tissue repair and their ability to home to the site of injury after systemic administration, several clinical trials for a wide range of diseases have been reported and are ongoing (Mahmood et al., 2003; Barry and Murphy, 2004; Steinert et al., 2012; Madrigal et al., 2014; Kim and Cho, 2015; Seebach et al., 2015). We extensively studied the FVV construct FV.U3-IL1RA-EGFP (NA1) compared to a FV.U3-EGFP (MD09) control vector in their ability to transduce mesenchymal cell lines and primary MSCs (**Figure 3**) and provide sustained long-term transgene expression in primary MSCs over a time period of 137 days which is noticeable (**Figure 4**). The cells were transduced in monolayer (passage 2), FACS sorted for EGFP and kept in 6-well plates without passaging over the time (**Figure 4**). In their state of replicative senescence, the cells maintained the transgene expression, without showing peculiar morphological changes over time (no enlarged cells), as reported by microscopy and FACS analysis. Remarkably, the IL1RA transgene expression persisted at levels around 50 ng/ml until the end of the monolayer culture experiment of more than 4 months (**Figure 4C**). The expression of the anti-inflammatory IL1RA might have had beneficial effects for inhibiting the senescent phenotype of MSCs under such culture conditions (**Figure 4C**).

Further it has been interestingly shown that transductions at late passages result in lower transgene expression levels compared to early passage transductions (McGinley et al., 2011). In our experiments, we were able to detect very high IL1RA amounts from aggregates with later passage cells (passage 7–9) that were transduced, FACS sorted and expanded (**Figure 6B**). The effect of the sorting procedure to the primary MSCs on the other hand resulted in lower IL1RA amount as compared to unsorted MSC pellets (**Figure 6B**), indicating that our sorting procedure might have stressed the cells.

The high levels of transgene expression in the pellet cultures following transduction with FV.U3-IL1RA-EGFP (**Figure 6**) compared to monolayer cultures (**Figures 2E**, **3B**, **4C**) might be attributed to the fact, that only 0.5 ml of media was used for each pellet, which allows strong accumulation of the secreted transgene product. Secondly, the high-density cell culture conditions might have triggered transgene expression, a phenomenon which was also seen in previous studies using adenovirus vectors in similar culture conditions (Steinert et al., 2009).

Because of their low frequency in bone-marrow, the *in vitro* expansion of MSCs prior to clinical use is necessary. Besides, as already mentioned, MSCs become senescent over time in culture, they are not further able to proliferate, show impaired functions and shortened telomers (Baxter et al., 2004; Bork et al., 2010; Duscher et al., 2014). The MSC donor age also significantly affects the rate of *in vitro* senescence in MSC, which also might have an effect on the transgene expression level and duration (Stenderup et al., 2003; Wagner et al., 2009). More detailed experiments to further study the long-term expression of (aged) MSCs as well as molecular senescence markers was beyond the scope of this study, but should be addressed in the future.

TGFβ1 supplementation in a defined serum-free medium in 3D aggregate cultures is routinely used to study the chondrogenic differentiation of MSCs *in vitro*, which has been characterized for cells from different species and also cultures with different biomaterials like alginate, agarose and fibrin alginate hydrogels have extensively been studied (Johnstone et al., 1998). Here we have chosen a biomaterial-free system, the 3D pellet culture system, to further verify IL1RA transgene expression in the MSC cell line TERT4 and primary MSCs (**Figures 5**, **6**).

The telomerase immortalized human MSC cell line TERT4 has been shown to bypass senescence, which has already been verified in several studies (Simonsen et al., 2002; Li et al., 2007; Bocker et al., 2008). Telomerase immortalization of TERT4 cells might also be responsible for the increasing levels of cell proliferation in pellet culture (**Figure 5E**), which is usually not seen in pellet cultures using primary MSCs (Johnstone et al., 1998; Steinert et al., 2009). Interestingly all groups, with exception of the IL1β group, showed a significantly increased cell proliferation especially in the last week of differentiation in the FV.U3-IL1RA-EGFP treatment group compared to the other groups (**Figure 5E**). However, the significance of this finding remains unclear to date and may arise upon further investigation.

Notably, we also detected higher GAG levels over the 21 days of differentiation in the TERT4 pellet groups, that expressed FV.IL1RA, compared to the untransduced chondrogenic positive control (gray bars, **Figure 5D**). This effect might be due to the IL1RA expression or FVV mediated, which also has to be further addressed in future experiments.

Although the IL1RA expression levels of EGFP+ FACS sorted MSCs were lower compared to the levels from unsorted transduced pellets, we performed our aggregate experiments with sorted cells (**Figures 5**, **6**). More experiments for an explicit picture of possible detrimental effects of the sorting procedure on the integrity of the cells should therefore be addressed in the future. We recently showed the feasibility of transduction and FACS sorting of primary rat synovial cells, that succesfully expressed the IL1RA transgene after reimplantation *in vivo* (Armbruster et al., 2014). Moreover, it has to be verified if DNA integration and the resultant transgene expression with high MOIs of FVVs, does adversely affect MSC plasticity, which has already been shown to be absent for lentiviral vectors (Van Damme et al., 2006; McGinley et al., 2011). Though, as we didn’t see chondrogenesis to be affected after FVV transduction within our experiments, other differentiation pathways might possibly be similarly unaffected by FVV transdcution itself.

After 21 days of chondrogenic differentiation we analyzed the pellets and found that the IL1β inhibited chondrogenesis could be equally rescued with FV.U3-IL1RA-EGFP transduced TERT4 cell line and primary MSC pellets regarding restored positive cartilage proteoglycan stainings (Alcian blue, **Figures 5C,D**). Correspondingly, the immunohistochemical analyses of FV.U3-IL1RA-EGFP transduced MSC pellets treated with IL1β showed a rescued Collagen II staining (**Figure 6E**). Although the staining was slightly weaker compared to the TGFβ positive controls, it appeared very homogenous with intense stainings being restricted to potentially hypertrophic areas (**Figure 6E**). Noteworthy we also found more homogenous Collagen II stainings in the positive control groups among our different MSC donors (data not shown), which might have been due to differences in the differentiation potential among the donors (Steinert et al., 2007; Mueller and Tuan, 2008; Yu and Kang, 2013).

The evaluation of chondrogensis in this work is limited to only one concentration of the chondrogenic inducer TGFβ1 (10 ng/ml), one concentration of the chondrogenic inhibitor IL1β (5 ng/ml), and only one dose of FV.U3-IL1RA-EGFP vector (100 MOI) which were used in the respective experiments using the TERT4 MSC cell line (**Figure 5**) and the primary MSCs (**Figure 6**), and only one type of 3D culture system (pellet culture). The inhibitory dose of IL1β of 5 ng/ml was used as this dose reflects a very high dose of this cytokine which is usually not seen as intraarticular concentration, even in severe arthritic or traumatic conditions (Martínez de Albornoz Torrente and Forriol, 2012; Schett et al., 2016). Furthermore, Wehling et al. (2009) studied the effects of IL1β in inhibiting chondrogenesis of primary MSCs in a dose-dependent manner on a molecular level, with the concentrations 1 and 10 ng/ml being most effective in the presence of 10 ng/ml TGFβ1, which argues for the choice of 10 ng/ml TGFβ1 as chondrogenic inducer and 5 ng/ml IL1β as chondrogenic inhibitor in our experimental set-up. Despite FVV dose-response experiments were perfomed using different types of FVV (**Figures 1–3**) for vector optimization, the analysis of different doses of FVV in blocking the inhibitory effects of 5 ng/ml IL1β on chondrogenesis would have been desirable. As our data on chondrogenesis is limited to protein level (**Figures 5**, **6**), future work on RNA level is mandatory, that comprises various aspects of chondrogenesis and inflammation including hypertrophy, osteogenic induction, and MMP regulation, among others.

## Conclusion

We constructed and verified several prototype foamyviral derived vector constructs and tested them on different mesenchymal cell types. The FVVs efficiently transduced primary human MSCs and a MSC cell line with high and sustainable transgene levels. In a 3D pellet culture model of chondrogenic differentiation the FVV mediated IL1RA expression was able to inhibit the effects of recombinant IL1β and to restore the chondrogenic phenotype of the aggregates. FVV might therefore be suitable to study and improve the outcome of MSC-based approaches for cartilage repair and merits further investigation to experimentally optimize such approaches in the future.

## Author Contributions

All authors have read and approved the manuscript and contributed to the study design, data analysis, interpretation of data and drafting, and revision of the manuscript. All data have been generated by NA, JK, CS, AS, and a data review committee (NA, JK, MW, CS, AS) analyzed and interpreted the data.

## Conflict of Interest Statement

The authors declare that the research was conducted in the absence of any commercial or financial relationships that could be construed as a potential conflict of interest.
